# Effects of hybrid comprehensive telerehabilitation on cardiopulmonary capacity in heart failure patients depending on diabetes mellitus: subanalysis of the TELEREH-HF randomized clinical trial

**DOI:** 10.1186/s12933-021-01292-9

**Published:** 2021-05-13

**Authors:** Renata Główczyńska, Ewa Piotrowicz, Dominika Szalewska, Ryszard Piotrowicz, Ilona Kowalik, Michael J. Pencina, Wojciech Zaręba, Maciej Banach, Piotr Orzechowski, Sławomir Pluta, Robert Irzmański, Zbigniew Kalarus, Grzegorz Opolski

**Affiliations:** 1 1st Chair and Department of Cardiology, Medical University of Warsaw, Warsaw, Poland; 2 Telecardiology Center, National Institute of Cardiology, Alpejska Str. 42, 04-828 Warsaw, Poland; 3 Clinic of Rehabilitation Medicine, Faculty of Health Sciences, Medical University of Gdańsk, Gdańsk, Poland; 4 National Institute of Cardiology, Warsaw, Poland; 5 Warsaw Academy of Medical Rehabilitation, Warsaw, Poland; 6 Duke University School of Medicine, Durham, NC USA; 7 University of Rochester Medical Center, Rochester, NY USA; 8 Department of Hypertension, Medical University of Łódź, Lodz, Poland; 9 Department of Cardiology, Congenital Heart Diseases and Electrotherapy, Silesian Center for Heart Diseases, Silesian Medical University, Zabrze, Poland; 10 Department of Internal Medicine and Cardiac Rehabilitation, Medical University of Łódź, Lodz, Poland

**Keywords:** Hybrid rehabilitation, Telerehabilitation, Heart failure, Exercise training, Diabetes mellitus

## Abstract

**Background:**

Type 2 diabetes mellitus (DM) is one of the most common comorbidities among patients with heart failure (HF) with reduced ejection fraction (HFrEF). There are limited data regarding efficacy of hybrid comprehensive telerehabilitation (HCTR) on cardiopulmonary exercise capacity in patients with HFrEF with versus those without diabetes.

**Aim:**

The aim of the present study was to analyze effects of 9-week HCTR in comparison to usual care on parameters of cardiopulmonary exercise capacity in HF patients according to history of DM.

**Methods:**

Clinically stable HF patients with left ventricular ejection fraction [LVEF] < 40% after a hospitalization due to worsening HF within past 6 months were enrolled in the TELEREH-HF (The TELEREHabilitation in Heart Failure Patients) trial and randomized to the HCTR or usual care (UC). Cardiopulmonary exercise tests (CPET) were performed on treadmill with an incremental workload according to the ramp protocol.

**Results:**

CPET was performed in 385 patients assigned to HCTR group: 129 (33.5%) had DM (HCTR-DM group) and 256 patients (66.5%) did not have DM (HCTR-nonDM group). Among 397 patients assigned to UC group who had CPET: 137 (34.5%) had DM (UC-DM group) and 260 patients (65.5%) did not have DM (UC-nonDM group). Among DM patients, differences in cardiopulmonary parameters from baseline to 9 weeks remained similar among HCTR and UC patients. In contrast, among patients without DM, HCTR was associated with greater 9-week changes than UC in exercise time, which resulted in a statistically significant interaction between patients with and without DM: difference in changes in exercise time between HCTR versus UC was 12.0 s [95% CI − 15.1, 39.1 s] in DM and 43.1 s [95% CI 24.0, 63.0 s] in non-DM, interaction p-value = 0.016. Furthermore, statistically significant differences in the effect of HCTR versus UC between DM and non-DM were observed in ventilation at rest: − 0.34 l/min [95% CI − 1.60, 0.91 l/min] in DM and 0.83 l/min [95% CI − 0.06, 1.73 l/min] in non-DM, interaction p value = 0.0496 and in VE/VCO_2_ slope: 1.52 [95% CI − 1.55, 4.59] for DM vs. − 1.44 [95% CI − 3.64, 0.77] for non-DM, interaction p value = 0.044.

**Conclusions:**

The benefits of hybrid comprehensive telerehabilitation versus usual care on the improvement of physical performance, ventilatory profile and gas exchange parameters were more pronounced in patients with HFrEF without DM as compared to patients with DM.

*Trial registration*: ClinicalTrials.gov Identifier: NCT02523560. Registered 3rd August 2015. https://clinicaltrials.gov/ct2/show/NCT02523560?term=NCT02523560&draw=2&rank=1. Other Study ID Numbers: STRATEGME1/233547/13/NCBR/2015

**Supplementary Information:**

The online version contains supplementary material available at 10.1186/s12933-021-01292-9.

## Introduction

Heart failure patients (HF) frequently present with comorbidities, which affect their prognosis and quality of life, including everyday activities and exercise performance. Type 2 diabetes mellitus (DM) is one of the most common comorbidities among patients with HF with reduced ejection fraction (HFrEF). Diabetes is associated with poorer functional status and worse prognosis in patients with HFrEF. Patients with DM often complain of fatigue and reduced exercise capacity [1].

Recent trials have shown that incidence of hospitalization due to HF were two-fold higher in diabetic patients compared with those without DM [2, 3]. Some trials and meta-analysis supported evidence that cardiac rehabilitation might be associated with modest reduction in HF hospitalization in the short term (up to 12 months) follow-up [4, 5]. Meta-analysis of 33 trials [6] confirmed the benefit of physical training on quality of life and exercise capacity. Based on numerous publications [7] and the latest European Society of Cardiology (ESC) guidelines, comprehensive cardiac rehabilitation has a strong evidence for improving cardiopulmonary exercise capacity and exercise tolerance in patients with HF [8]. The last 2020 Sports Cardiology ESC guidelines underlined that exercise-based cardiac rehabilitation is recommended in all stable individuals [8] to improve exercise capacity, quality of life, and to reduce the frequency of hospital readmission [7]. Also, intensive lifestyle intervention with focusing on increased physical activity is recommended by American Diabetes Association for diabetic patients [9, 10].

Cardiopulmonary exercise testing (CPET) has proven to be useful for quantifying aerobic capacity and is valuable for identifying exercise tolerance in patients with cardiac diseases [11, 12], and to assess functional capacity, exercise-induced arrhythmias, and haemodynamic abnormalities [13]. According to the current ESC guidelines for the diagnosis and treatment of acute and chronic heart failure, CPET should be considered to optimize exercise training (class IIa/level of evidence C) [14]. Cardiopulmonary exercise testing allows safe, non-invasive, and reliable assessment of overall fitness which incorporates cardiovascular, pulmonary and musculoskeletal responses to exercise [15, 16]. It is a method combining continuous expired gases analysis, electrocardiography, blood pressure measurements and oxygen saturation monitoring during gradually increasing workload [17]. This method provides breath-by-breath gas exchange measures [18] of variables such as O_2_ uptake (VO_2_), carbon dioxide output (VCO_2_), and ventilation (VE), which are used to derive various other gas exchange patterns that reflect organ-specific maladaptive responses to exercise [16].

The expert group recommends assessment of the clinical impact of CPET as a high research priority [19]. Since significant proportion of HF patients presents with DM there is interest in assessing effects of cardiac rehabilitation in DM vs. non-DM patients. Our post-hoc analysis from the Telerehabilitation in Heart Failure Patients (TELEREH-HF) randomized clinical trial [20, 21] provides interesting insight into differences in response to rehabilitation in patients with and without DM.

The aim of the present subanalysis from the TELEREH-HF trial [20, 21] was to compare cardiopulmonary exercise capacity in HFrEF patients undergoing hybrid comprehensive telerehabilitation (HCTR) or usual care (UC) in patients with and without diabetes.

## Methods

The design and main results of the TELEREH-HF trial have been presented already elsewhere [20, 21]. The TELEREH-HF study was a randomized, multi-center, prospective, open-label, parallel group, controlled trial, which enrolled clinically stable HF patients [New York Heart Association (NYHA) class I, II or III] with left ventricular ejection fraction (LVEF) ≤ 40% after a hospitalization due to worsening HF within 6 months prior to randomization.

All HCTR patients from both subgroups (with and without DM), underwent a 9-week HCTR program consisting of two stages: an initial stage (1 week) conducted in hospital and a basic home-based stage (8 weeks) with HCTR performed five times weekly (see Additional file 1: Table S1). Patients in UC were managed according to routine standard of care.

HCTR was conducted by a medical team (physicians, physiotherapists, nurses and psychologist). The monitoring system was composed of a remote device for tele-ECG-monitoring and supervised exercise training (the telerehabilitation set), mobile phone, and a monitoring center.

The research protocol was registered in a clinical database (ClinicalTrials.gov NCT02523560) and followed the ethical guidelines of Declaration of Helsinki. The ethics committee approved the research protocol. Informed consent was obtained from patients.

### Cardiopulmonary exercise testing

In this subanalysis, data on cardiopulmonary exercise capacity in HF patients with and without DM undergoing HCTR or UC were compared. Functional capacity parameters were evaluated using symptom-limited CPET on treadmill (Schiller MTM-1500 med) with an incremental workload according to the ramp protocol equal for all patients. The initial treadmill speed and slope were 1.5 mph and 1.5%, respectively. In every 30 s, treadmill speed and slope were increased by 0.5 mph and 0.5%, respectively. Maximal exercise was defined as the respiratory exchange ratio (RER) ≥ 1 or maximum fatigue according to the Borg scale (fatigue level 14–16 on a 20-point scale). Oxygen uptake was analyzed on a continuous breath-by-breath basis. Obtained peak oxygen uptake (VO_2_ peak) values are presented per kilogram of mass body per minute (ml/kg/min) and as a percentage of normal for each patient called percent-predicted VO_2_ (pVO_2_% N) with gender, age, weight and height according to the criteria developed by Wasserman.

Ventilation and gas exchange parameters acquired from CPET comprised exercise duration time, carbon dioxide production (VCO_2_), minute ventilation (VE), breathing rate and slope of ventilatory equivalent for carbon dioxide (VE/VCO_2_ slope). To determine ventilatory anaerobic threshold (VAT) the V-slope method of plotting VCO_2_ against VO_2_ was applied.

The test was continued until symptoms indicating the need to discontinue the test appeared, in accordance with ESC recommendations [22].

### Statistical analysis

Results are expressed as means ± SD (baseline characteristics) or means and 95% confidence limits (difference between 9-week value and baseline value) for continuous variables or counts and percentages for categorical variables. Comparisons between groups at baseline characteristics were made using chi-square test of independence or Fisher’s exact test (when the number of expected events was less than 5), Cochran Mantel–Haenszel test or Student’s t-tests (or Satterthwaite’s method), as appropriate. The differences in changes over time between the groups were compared using Two-Way Analysis of Variance with baseline measurement as covariance. Interaction was tested. A two-sided p < 0.05 was considered statistically significant. All analyses were performed using SAS statistical software version 9.4 (SAS Institute, Inc., Cary, North Carolina, USA).

## Results

### Baseline characteristics

**Fig. 1:**
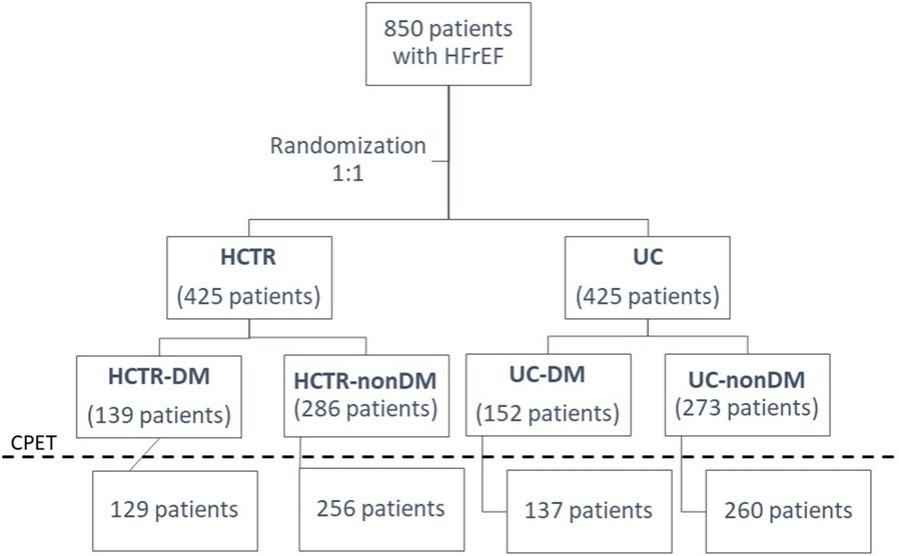
Study design. *HCTR-DM* patients in hybrid comprehensive telerehabilitation arm with heart failure and diabetes, *HCTR-nonDM* patients in hybrid comprehensive telerehabilitation arm with heart failure and without diabetes, *HFrEF* heart failure with reduced ejection fraction, *UC-DM* patients in usual care arm with heart failure and diabetes, *UC-nonDM* patients in usual care arm with heart failure and without diabetes

Between June 8th, 2015 and June 28th, 2017, 850 eligible patients were randomized in a 1:1 ratio to either HCTR (HCTR group) or to UC (UC group). 425 patients of either sex with HF with reduced ejection fraction (HFrEF), enrolled in the TELEREH-HF trial with no contraindication to training and able to undergo HCTR were randomized to HCTR arm (Additional file 1: Table S2). Among 850 enrolled patients, 291 (34.2%) patients had DM. Among 425 patients assigned to HCTR group, CPET was performed twice before and after telerehabilitation programme in 385 patients: 129 (33.5%) had DM (HCTR-DM group) and 256 patients (66.5%) did not have DM (HCTR-nonDM group). Among 425 patients assigned to UC group CPET was done twice in 397 patients: 137 (34.5%) had DM (UC-DM group) and 260 patients (65.5%) did not have DM (UC-nonDM group). List of the reason for lack of both CPETs and baseline characteristics of 68 patients comparing to those included in our paper are presented in the Additional file 1: Table S3. The study flow diagram is presented in Fig. 1. Study arms HCTR vs UC were not significantly different by randomization in terms of demographic data, baseline clinical parameters, and treatment. The baseline characteristics of the cohort at randomization are presented in Table 1.

**Table 1:** Baseline characteristics of studied patients with and without diabetes by randomization

	DM, n = 266			nonDM, n = 516		
	HCTR-DM n = 129	UC-DM n = 137	p1	HCTR-nonDM n = 256	UC-nonDM n = 260	p2
Males, n (%)	120 (93.0)	125 (91.2)	0.590	225 (87.9)	228 (87.7)	0.945
Age (years), mean ± SD	65.1 ± 8.1	63.4 ± 7.9	0.090	60.6 ± 11.7	61.1 ± 11.2	0.668
BMI (kg/m^2^), mean ± SD	30.0 ± 5.1	31.1 ± 4.6	0.068	28.2 ± 5.0	28.0 ± 4.4	0.711
Left ventricular ejection fraction (%), mean ± SD	30.8 ± 6.2	29.9 ± 7.0	0.286	31.1 ± 7.2	30.7 ± 7.0	0.491
Atrial fibrillation or atrial flutter, n (%)	28 (21.7%)	31 (22.6)	0.856	45 (17.6)	41 (15.8)	0.581
Etiology of heart failure, n (%)						
Ischaemic	95 (73.6)	95 (69.3)	0.438	158 (61.7)	158 (60.8)	0.825
Non-ischeamic	34 (26.4)	42 (30.7)		98 (38.3)	102 (39.2)	
Previous medical history, n (%)						
Coronary artery disease	94 (72.9)	93 (67.9)	0.374	159 (62.1)	156 (60.0)	0.623
Myocardial infarction	84 (65.1)	84 (61.3)	0.521	142 (55.5)	137 (52.7)	0.527
Angioplasty	65 (50.4)	69 (50.4)	0.997	119 (46.5)	111 (42.7)	0.386
Coronary artery bypass grafting	30 (23.3)	27 (19.7)	0.481	28 (10.9)	37 (14.2)	0.260
Hypertension	93 (72.1)	103 (75.2)	0.567	134 (52.3)	152 (58.5)	0.162
Stroke	8 (6.2)	16 (11.7)	0.119	13 (5.1)	14 (5.4)	0.876
Chronic kidney disease	36 (27.9)	36 (26.3)	0.765	34 (13.3)	25 (9.6)	0.191
Hyperlipidemia	67 (51.9)	58 (42.3)	0.117	124 (48.4)	115 (44.2)	0.338
Functional status						
NYHA I, n (%)	7 (5.4)	11 (8.0)	0.275	44 (17.2)	38 (14.6)	0.613
NYHA II, n (%)	91 (70.5)	84 (61.3)		181 (70.7)	185 (71.2)	
NYHA III, n (%)	31 (24.1)	42 (30.7)		31 (12.1)	37 (14.2)	
Treatment						
Beta-blocker	128 (99.2)	136 (99.3)	0.966	241 (94.1)	253 (97.3)	0.075
ACEI/ARB	119 (92.2)	127 (92.7)	0.889	240 (93.7)	245 (94.2)	0.818
Digoxin	21 (16.3)	26 (19.0)	0.564	27 (10.5)	23 (8.8)	0.514
Loop diuretics	108 (83.7)	117 (85.4)	0.704	174 (68.0)	192 (73.8)	0.142
Spironolactone/eplerenone	104 (80.6)	107 (78.1)	0.612	215 (84.0)	217 (83.5)	0.872
Aspirin/clopidogrel	74 (57.4)	77 (56.2)	0.849	145 (56.6)	147 (56.5)	0.981
Anticoagulants	43 (33.3)	50 (36.5)	0.589	70 (27.3)	71 (27.3)	0.993
Statins	110 (85.3)	122 (89.1)	0.356	205 (80.1)	202 (77.7)	0.507
CIEDs	107 (82.9)	116 (84.7)	0.702	197 (76.9)	206 (79.2)	0.532
Implantable cardioverter-defibrillator	61 (57.0)	70 (60.3)	0.815	128 (65.0)	137 (66.5)	0.905
CRT-P	1 (0.9)	0 (0)		3 (1.5)	4 (1.9)	
CRT-D	44 (41.1)	45 (38.8)		64 (32.5)	62 (30.1)	

*NYHA* New York Heart Association class, *ACEI* angiotensin converting enzyme inhibitors, *ARB* angiotensin receptor blockers, *CIEDs* cardiovascular implantable electronic devices (including pacemakers, implantable cardioverter defibrillators, cardiac resynchronization therapy and cardiac resynchronization therapy with cardioverter-defibrillator), *CRT-P* cardiac resynchronization therapy, *CRT-D* cardiac resynchronization therapy and cardioverter-defibrillator, *DM* diabetes mellitus, *HCTR-DM* patients in hybrid comprehensive telerehabilitation arm with heart failure and diabetes, *HCTR-nonDM* patients in hybrid comprehensive telerehabilitation arm with heart failure and without diabetes, *UC-DM* patients in usual care arm with heart failure and diabetes, *UC-nonDM* patients in usual care arm with heart failure and without diabetes

p1—p value for HCTR-DM vs. UC-DM, p2—p value for HCTR-nonDM vs. UC-nonDM

### Cardiopulmonary capacity

There was no difference in CPET parameters between HCTR and UC arms at the time of randomization (Table 2). But at the time of randomization the groups with and without DM differed in terms of CPET parameters. The baseline exercise time was longer in nonDM patients than DM patients in both HCTR arm (420 ± 185 vs. 338 ± 163 [s], p < 0.001) and in UC arm (407 ± 188 vs. 327 ± 163 [s], p < 0.001). At randomization VO_2_ peak was greater in patients without DM as compared to those with DM in HCTR group (18.1 ± 5.8 vs. 15.3 ± 4.5 [ml/min/kg], p < 0.001) and in UC group (17.7 ± 6.3 vs. 15.1 ± 5.1 [ml/min/kg], p < 0.001). Percent-predicted VO_2_ (%) was higher at randomization among patients without DM than in patients with DM both in HCTR arm (59.6 ± 20.7 vs. 49.4 ± 17.9 [%], p < 0.001) and UC arm (58.7 ± 22.0 vs. 47.0 ± 16.5 [%], p < 0.001). At the time of randomization ventilatory anaerobic threshold was greater in nonDM patients as compared to DM patients, either after HCTR (15.9 ± 5.7 vs. 13.6 ± 4.5 [ml/kg/min], < 0.001) and UC (15.6 ± 5.8 vs. 13.9 ± 5.5 [ml/kg/min], p = 0.029). Difference in VO_2_ peak was higher in HCTR-nonDM group when compared to UC-nonDM (1.33 [95% CI 0.92; 1.74] vs. 0.07 [95% CI − 0.33; 0.48] ml/min/kg, p < 0.001). Only in UC arm VE/VCO_2_ slope was lower in nonDM patients than in DM patients (29.3 ± 11.0 vs. 31.8 ± 10.5, p = 0.033). Other important CPET parameters did not differ between patients with and without DM at the time of randomization.

**Table 2:** Baseline parameters of cardiopulmonary capacity

CPET parameters	DM (n = 266)			nonDM (n = 516)		
	HCTR-DM n = 129	UC-DM n = 137	p1	HCTR-nonDM n = 256	UC-nonDM n = 260	p2
Exercise time (s)	338 ± 163	327 ± 163	0.602	420 ± 185	407 ± 188	0.427
VO_2_peak (ml/min/kg)	15.3 ± 4.5	15.1 ± 5.1	0.821	18.1 ± 5.8	17.7 ± 6.3	0.417
VCO_2_peak (l/min)	1.29 ± 0.55	1.38 ± 0.58	0.193	1.56 ± 0.67	1.51 ± 0.69	0.389
Percent-predicted VO_2_ (%)	49.4 ± 17.9	47.0 ± 16.5	0.255	59.6 ± 20.7	58.7 ± 22.0	0.638
VAT (ml/kg/min):	13.6 ± 4.5	13.9 ± 5.5	0.634	15.9 ± 5.7	15.6 ± 5.8	0.651
Ventilation at rest (l/min)	13.0 ± 4.0	13.3 ± 4.6	0.568	12.8 ± 5.5	12.9 ± 4.1	0.897
Ventilation on peak exercise (l/min)	44.9 ± 16.0	47.5 ± 15.8	0.177	51.7 ± 18.8	50.4 ± 19.3	0.419
Breathing rate at rest(1/min)	18.8 ± 4.6	19.0 ± 4.9	0.684	18.7 ± 4.6	19.1 ± 5.1	0.372
Breathing rate on peak exercise (1/min)	28.8 ± 5.9	29.0 ± 5.7	0.826	29.6 ± 6.5	29.8 ± 6.8	0.731
VE/VO_2_ slope	28.4 ± 10.0	31.7 ± 13.4	0.029	31.0 ± 10.9	31.0 ± 11.1	0.994
VE/VCO_2_ slope	32.1 ± 9.7	31.8 ± 10.5	0.778	30.3 ± 9.7	29.3 ± 11.0	0.293

*CPET* Cardiopulmonary Exercise Testing, *HCTR-DM* patients in hybrid comprehensive telerehabilitation arm with heart failure and diabetes, *HCTR-nonDM* patients in hybrid comprehensive telerehabilitation arm with heart failure and without diabetes, *VO*_*2*_* peak* peak oxygen uptake, *UC-DM* patients in usual care arm with heart failure and diabetes, *UC-nonDM* patients in usual care arm with heart failure and without diabetes, *VAT* ventilatory anaerobic threshold, *VE/VCO*_*2*_* slope* slope of ventilatory equivalent for carbon dioxide

p1—p value for HCTR-DM vs. UC-DM, p2—p value for HCTR-nonDM vs. UC-nonDM

As presented in Table 3, among DM patients, cardiopulmonary parameters remained similar from baseline to 9 weeks after telerehabilitation or observation. In HCTR-DM group, VAT was achieved in 64 patients (55.2%) at baseline and in 70 patients (60.3%, p = 0.601) after telerehabilitation programme. In UC-DM group, VAT was achieved in 67 patients (55.8%) at baseline and in 64 patients (53.3%, p = 0.601) after 9-week UC.

**Table 3:** Changes from baseline to 9 weeks in parameters of cardiopulmonary capacity in patients with diabetes mellitus (adjusted for baseline)

CPET parameters	With DM			
	Δ 9 week–baseline [95% CI]		Difference [95% CI]	p
	HCTR-DM	UC-DM		
Exercise time (s)	35.9 [21.0; 50.8]	23.9 [9.4; 38.4]	12.0 [− 15.1; 39.1]	0.666
VO_2_ peak (ml/min/kg)	0.40 [− 0.18; 0.98]	− 0.15 [− 0.72; 0.41]	0.55 [− 0.50; 1.61]	0.529
Percent-predicted VO_2_ (%)	1.69 [− 0.49; 3.86]	− 2.04 [− 4.16; 0.09]	3.73 [− 0.22; 7.66]	0.072
VAT (ml/kg/min)	− 0.22 [− 1.06; 0.62]	0.16 [− 0.73; 1.05]	− 0.38 [− 1.98; 1.21]	0.926
Ventilation at rest (l/min)	− 0.38 [− 1.07; 0.30]	− 0.04 [− 0.70; 0.63]	− 0.34 [− 1.60; 0.91]	0.892
Ventilation on peak exercise (l/min)	1.54 [− 0.44; 3.52]	0.54 [− 1.37; 2.46]	1.00 [− 2.60; 4.60]	0.891
Breathing rate at rest (1/min)	0.11 [− 0.61; 0.83]	0.30 [− 0.40; 0.99]	− 0.19 [− 1.50; 1.12]	0.983
Breathing rate on peak exercise (1/min)	0.77 [− 0.02; 1.55]	− 0.25 [− 1.01; 0.51]	1.02 [− 0.42; 2.45]	0.263
VE/VCO_2_ slope	1.42 [− 0.25; 3.09]	− 0.10 [− 1.74; 1.54]	1.52 [− 1.55; 4.59]	0.579

*CI* confidence interval, *CPET* Cardiopulmonary Exercise Testing, *HCTR-DM* patients in hybrid comprehensive telerehabilitation arm with heart failure and diabetes, *HCTR-nonDM* patients in hybrid comprehensive telerehabilitation arm with heart failure and without diabetes, *VO*_*2*_* peak* peak oxygen uptake, *UC-DM* patients in usual care arm with heart failure and diabetes, *UC-nonDM* patients in usual care arm with heart failure and without diabetes, *VAT* ventilatory anaerobic threshold, *VE/VCO*_*2*_* slope* slope of ventilatory equivalent for carbon dioxide

Among patients without DM, exercise time increased significantly more in HCTR as compared to UC—change after 9 weeks from baseline: 56.7 s [95% CI 46.1, 67.3 s] vs. 13.6 s [95% CI 3.2, 24.1], p-value < 0.001. In HCTR-nonDM group patients, VAT was reached more often after 9-week telerehabilitation (in 149 patients [68.7%] vs. 129 patients [61.1%], p = 0.042, after 9 weeks vs. at baseline, respectively). In UC-nonDM group, VAT was reached in 129 patients (58.9%) at baseline and in 140 patients (63.9%, p = 0.116) after 9-week UC. But not significant changes in VAT were noticed both in patients with and without DM. All alterations from baseline to 9 weeks in parameters of cardiopulmonary capacity in patients without diabetes mellitus are shown in Table 4.

**Table 4:** Changes from baseline to 9 weeks in parameters of cardiopulmonary capacity in patients without diabetes mellitus (adjusted for baseline)

CPET parameters	Without DM			
	Δ 9 week–baseline [95% CI]		Difference [95% CI]	p
	HCTR-nonDM	UC-nonDM		
Exercise time (s)	56.7 [46.1; 67.3]	13.6 [3.2; 24.1]	43.1 [24.0; 63.0]	< 0.001
VO_2_ peak (ml/min/kg)	1.33 [0.92; 1.74]	0.07 [− 0.33; 0.48]	1.26 [0.50; 2.02]	< 0.001
Percent-predicted VO_2_ (%)	4.02 [2.48; 5.56]	− 0.07 [− 1.60; 1.46]	4.09 [1.25; 6.92]	0.001
VAT (ml/kg/min)	1.45 [0.85; 2.06]	0.91 [0.30; 1.52]	0.54 [− 0.58; 1.67]	0.601
Ventilation at rest (l/min)	0.82 [0.34; 1.31]	− 0.01 [− 0.49; 0.47]	0.83 [− 0.06; 1.73]	0.082
Ventilation on peak exercise (l/min)	4.00 [2.59; 5.40]	− 0.16 [− 1.55; 1.23]	4.16 [1.57; 6.75]	< 0.001
Breathing rate at rest (1/min)	0.42 [− 0.10; 0.93]	− 0.17 [− 0.68; 0.33]	0.59 [− 0.35; 1.53]	0.370
Breathing rate on peak exercise (1/min)	1.04 [0.48; 1.60]	− 0.24 [− 0.79; 0.32]	1.28 [0.25; 2.31]	0.008
VE/VCO_2_ slope	− 1.11 [− 2.30; 0.08]	0.33 [− 0.86; 1.51]	− 1.44 [− 3.64; 0.77]	0.336

*CI* confidence interval, *CPET* cardiopulmonary exercise testing, *HCTR-DM* patients in hybrid comprehensive telerehabilitation arm with heart failure and diabetes, *HCTR-nonDM* patients in hybrid comprehensive telerehabilitation arm with heart failure and without diabetes, *VO*_*2*_* peak* peak oxygen uptake, *UC-DM* patients in usual care arm with heart failure and diabetes, *UC-nonDM* patients in usual care arm with heart failure and without diabetes, *VAT* ventilatory anaerobic threshold, *VE/VCO*_*2*_* slope* slope of ventilatory equivalent for carbon dioxide

**Fig. 2:**
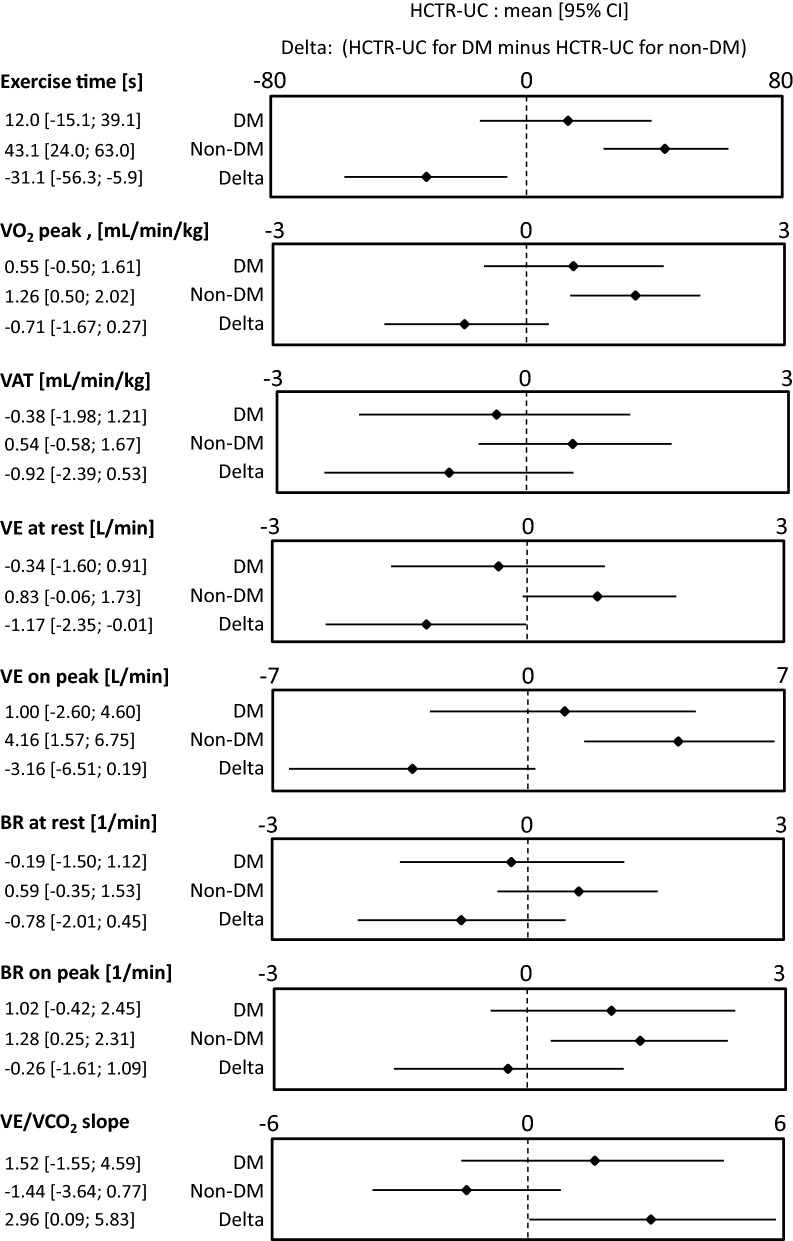
Forest plots of changes in parameters of main cardiopulmonary capacity in patients with and without diabetes. *delta* alteration in CPET parameters between baseline and 9-week based on formula: HCTR-UC for DM minus HCTR-UC for non-DM, *DM* patients with diabetes, *nonDM* patients without diabetes, *VO*_*2*_* peak* peak oxygen uptake, *VAT* ventilatory anaerobic threshold, *VE/VCO*_*2*_* slope* slope of ventilatory equivalent for carbon dioxide

The distinct differences were noticed between patients with and without DM after HCTR vs. UC in CPET parameters such as exercise time (12.0 s [95% CI − 15.1, 39.1 s] vs. 43.1 s [95% CI 24.0, 63.0 s], respectively, interaction p-value = 0.016), ventilation at rest (− 0.34 l/min [95% CI − 1.60, 0.91 l/min] vs. 0.83 l/min [95% CI − 0.06, 1.73 l/min], respectively, interaction p value = 0.0496) and VE/VCO_2_ slope (1.52 [95% CI − 1.55, 4.59] vs. − 1.44 [95% CI − 3.64, 0.77], respectively, interaction p value = 0.044). All differences in CPET parameters from baseline to 9 weeks are presented on Fig. 2.

Our study also provided evidence that both HCTR and UC are similarly safe in DM as in nonDM without significant adverse events, therefore participation in rehabilitation of HF patients with diabetes should be encouraged.

## Discussion

Our study is the first to present the characteristics of cardiac performance parameters in large group of HF patients with vs. without diabetes undergoing cardiac rehabilitation emplying innovative HCTR vs. UC. We have to emphasize that the randomization in the TELEREH-HF trial was done to compare HFrEF patients undergoing HCTR vs. UC and analyses of DM vs. nonDM groups is a secondary post-hoc analysis. The patients with and without DM were initially different in their clinical characteristics (Additional file 1: Table S4) and the CPET dissimilarities (Additional file 1: Table S5) were therefore consequences of its metabolic disparities. Till now, there were no large available data on the CPET in patients with HFrEF and DM. Therefore, it seems that our study is the first to present the characteristics of cardiac performance parameters in this specific but numerous group of patients.

In this substudy of the TELEREH-HF clinical trial, we analyzed effects of 9-week HCTR in comparison to usual care on parameters of cardiopulmonary exercise capacity in HF patients according to history of DM. Patients with DM accounted for about one-third of studied patients. Among DM patients, differences in cardiopulmonary parameters from baseline to 9 weeks remained similar among HCTR and UC patients. In contrast, among patients without DM, HCTR was associated with greater 9-week changes than UC in exercise time, in ventilation at rest and in VE/VCO2 slope. The benefits of HCTR versus UC on the improvement of physical performance, ventilatory profile and gas exchange parameters were found to be more pronounced in patients with HFrEF without DM as compared to patients with DM.

Measured VO_2_ during a maximal symptom-limited CPET is the most objective method to assess functional capacity and consists of the following components such as maximal heart rhythm, stroke volume, the net oxygen extraction of the peripheral tissues. Peak oxygen consumption is an important predictor of prognosis in HF patients [23]. In patients with HF, the important prognostic value of a reduced peak VO_2_ has been studied in detail to identify patients at higher risk.

In one recent trial, among many CPET variables assessed in patients with HF, VO_2_ peak, percent predicted VO_2_, and exercise duration had the strongest ability to predict mortality in HFrEF [24]. In line with that, improvements of cardiopulmonary capacity determined by VO_2_ peak, percent predicted VO_2_ and distance, was observed in our study in majority of patients after HCTR. An aerobic exercise training has been recommended as non-pharmacological treatment for patients with HFrEF. As we described in the previous article of TELEREH-HF trial, the HCTR intervention was effective at 9 weeks, significantly improving VO_2_ peak (0.95 [95% CI 0.65–1.26] ml/kg/min vs. 0.00 [95% CI − 0.31 to 0.30] ml/kg/min; p < 0.001) [20]. EMPA-TROPISM Trial with 84 HFrEF patients demonstrated that empagliflozin was associated with significant improvements in peak VO_2_ (1.1 ± 2.6 ml/min/kg vs. − 0.5 ± 1.9 ml/min/kg for empagliflozin vs. placebo; p = 0.017) [25].

Some studies have found decline in VO_2_ peak of 20–30% in population of diabetic patients without cardiovascular disorders. Authors of the recent paper tried to explain impaired cardiopulmonary capacity in diabetic patients by two underlying mechanism [26, 27]. It is the first one, called myocardiogenic determinants, an insufficient cardiac function reduces muscle perfusion, and thus determines insufficient muscle energy production and strength. In the hypothesis with skeletal myogenic determinants, reduced VO_2_ peak and peripheral oxygen extraction are consequence of slower muscle blood flow adjustment and early stimulation of the muscle metaboreflex [28]. In study by Ishihara et al. performed on group of 69 HF patients (with preserved or moderate EF) VO_2_ peak was lower in 14 DM patients when compared to 55 patients without DM (13.0 ± 2.2 vs. 14.9 ± 4.4 ml/kg/min, p < 0.05, respectively) [29].

In the recent study, a multivariate analysis showed that the history of DM was an independent predictor of lower VO_2_ peak in HF patients, but the impact on exercise capacity was dependent on the systolic dysfunction [30]. This is in line with the results of our study, where VO_2_ peak was lower in DM patients with HFrEF. Our baseline data of cardiopulmonary exercise profile are consistent with recent data from the study concerning exercise capacity in patients with HFrEF and metabolic syndrome [31] as well as from the relatively small study with HFrEF and DM [25].

Oxygen consumption at the ventilatory threshold (VAT) is another measurement of O_2_ uptake that provides valuable information at submaximal exercise. It represents upper border of workload which can be sustained for a prolonged period of exercise [32]. But, in the most advanced stages of HF a clear VAT is often not identifiable, which is indices for poor prognosis [33]. Not only lower VO_2_ peak but also reduced VAT was noticed in the diabetic patients with both HFrEF and HF with preserved ejection fraction [30]. In our work, only in HCTR-nonDM, VAT was reached more often. It cannot be interpreted as a weak cardiac telerehabilitation effect in the group of patients with DM, but this result might be a consequence of too short telerehabilitation duration. Maybe to gain better results in subpopulation in patients with HFrEF and DM, there is a need for prolongation of cardiac rehabilitation.

Other ventilatory parameters, such as increased ventilatory efficiency (expressed as ventilation to carbon dioxide production, VE/VCO_2_ slope) during exercise have been observed and associated with poor prognosis in HF [34, 35]. During exercise, efficient pulmonary gas exchange is characterized by uniform matching of lung ventilation with perfusion. By contrast, mismatching is marked by inefficient pulmonary gas exchange, requiring increased ventilation for a given CO_2_ production [36]. Inefficient ventilatory response to exercise in heart disease is multifactorial. Previous investigations found ventilation/perfusion inequalities during exercise in HF patients [37]. In fact, this finding suggests pathologically high ventilation/perfusion mismatching defined as a reduced or absent perfusion in well ventilated lung.

Notably, substantial evidence demonstrated that ventilatory inefficiency is an independent powerful prognostic marker for cardiac mortality or hospitalization in HF patients. VE/VCO_2_ slope broadly reflects disease severity in population of HF patients. VE/VCO_2_ slope as a continuous variable may be useful as a predictor for major cardiac events in patients with HF. VE/VCO_2_ slope can provide prognostic information beyond VO_2_ peak [38]. The inappropriate ratio of hyperventilation over CO_2_ production may reflect autonomic dysfunction and altered central control of breathing, but there are a few physiological mechanism of that phenomenon in DM. Steeper VE/VCO_2_ slope was observed in diabetic patients with cardiac autonomic dysfunction [39]. In recent study, there was present only a trend toward improvement in the VE/VCO_2_ in the empagliflozin versus placebo group (− 1.2 ± 3.4 vs. 0.5 ± 3.9, respectively; p = 0.09) [25].

Special focus was dedicated to the role of aerobic exercise training in improving the indices of ventilatory efficiency among patients with HFrEF, as well as to the underlying mechanisms involved. We observed improvement in ventilatory drive after HCTR. In literature, there is scarce data regarding ventilatory and capacity profile of CPET parameters among diabetic patients. Albeit a specific derangement of the alveolar capillary membrane in diabetic patients has been suggested [40].

In contrary to our work, in population without HF, DM was associated with greater improvements in response to exercise programme, when compared to the non-diabetic controls undergoing the same training [41]. There is literature bias, if exercise training may be effective in increasing VO_2_ peak in patients with DM. The physiological explanation for that observation is that the muscle deconditioning is not the only one mechanism of altered cardiopulmonary capacity, as mentioned previously. As it is well known effort has a positive effect on the course of diabetes in terms of its control.

As we described in previous substudy of TELEREH-HF trial, HCTR might have had beneficial effects on cardiopulmonary exercise test time after 9 weeks in patients with ischaemic HF, but it not met statistical significance from that observed among non-ischaemic HF patients [42]. Although, in our study there were higher percentage of ischaemic etiology among DM patients (Additional file 1: Table S4), we did not observed improvement in profile of cardiopulmonary capacity in patients with HFrEF and DM.

Less effective 9-week comprehensive telerehabilitation program in patients with DM may indicate the need to diversify training in these groups. This hypothesis fits nicely with the fashionable and right concept of ‘tailored training’ and ‘personalized therapy’ for some patients’ party, in this case DM patients. Initial conclusions may lead to statement consistent with European recommendations for exercise programme in DM populations. According to the 2020 ESC Guidelines on sports cardiology [8] and exercise in patients with CVD the ideal exercise programme in subjects with diabetes is daily exercise of at least moderate intensity. Among recommended activity there should we brisk walking, for at least 30 min, resistance training for 15 min on most days and lighter-intensity activities for at least 30 min. In case of microvascular complications due to diabetes those activities might be supplemented by flexibility and balance exercise. As we know, rehabilitation programme combining aerobic and resistance training [43] has been shown to be superior in diabetic population, whereas the effects on the outcomes are unproven [44]. Patients with DM are more demanding and need longer protocols and more intensive aerobic exercise training programme with health life counselling [45].

The optimal duration, volume and intensity of comprehensive cardiac rehabilitation programme in patients with cardiac comorbidities and DM vary with respect to the training goals. It should be definitely personalized. Patients severely detrained and with HF should start exercising at low intensity, and then with incremental workload. High-volume and moderate intensity training are recommended for improving body composition and CV risk factors. High intensity interval programme can be helpful for improvements in exercise capacity and glycaemic control, with goal of better prognosis. If cardiorespiratory fitness is a target for rehabilitation programme in patients with DM, combined aerobic and resistance training is preferred [45]. Lifestyle education and dietary counselling are crucial during comprehensive cardiac rehabilitation programme. In order to improve adherence, patient with DM should be engaged in home-based rehabilitation programme [46]. Nevertheless, rehabilitation programmes based on exercise in population with HFrEF and DM are associated with the higher risk of post-exercise hypotension, arrhythmias and HF decompensation.

Because of the study design with randomization of eligible patients in a 1:1 ratio (block size of 2, stratified by site) to HCTR or UC group, we decided to present and discuss reliable data in four subgroups (HCTR-DM, HCTR-nonDM, UC-DM, UC-nonDM). This study was not randomizing patients with vs. without diabetes and the analyses presented are post-hoc analyses, nevertheless, based on large well-balanced patient subgroups with and without diabetes.

## Conclusions

The beneficial effect of hybrid comprehensive telerehabilitation versus usual care on the improvement of physical performance, ventilatory profile and gas exchange parameters in cardiopulmonary exercise test was observed only in patients with HFrEF without DM.

## Supplementary Information

**Additional file 1: Table S1.** TELEREH-HF Exercise Training Model [21]. **Table S2.** Inclusion and Exclusion Criteria for TELEREH-HF trial [20, 21]. **Table S3.** Baseline characteristics of excluded vs studied patients. **Table S4.** Baseline characteristics of studied patients with and without diabetes. **Table S5.** Baseline parameters of cardiopulmonary capacity.

## Data Availability

An independent data safety monitoring board reviewed patient data, and a clinical end point committee, blinded to treatment allocation, was appointed to adjudicate deaths and hospitalizations. The study followed the Consolidated Standards of Reporting Trials guidelines.

## Abbreviations

CPET: Cardiopulmonary exercise test
DM: Diabetes mellitus
HCTR: Hybrid comprehensive telerehabilitation
HCTR-nonDM: Hybrid comprehensive telerehabilitation group without diabetes
HCTR-DM: Hybrid comprehensive telerehabilitation group with diabetes
HF: Heart failure
HFrEF: Heart failure with reduced ejection fraction
VAT: Ventilatory threshold
VCO_2_: Carbon dioxide production
VE: Minute ventilation
VE/VCO_2_slope: Slope of ventilatory equivalent for carbon dioxide or “ventilatory efficiency”
VO_2_: Oxygen uptake
VO_2_peak: Peak oxygen uptake
UC: Usual care
UC-nonDM: Usual care group with diabetes
UC-DM: Usual care group without diabetes
