# Supplementary material for: Effects of hybrid comprehensive telerehabilitation on cardiopulmonary capacity in heart failure patients depending on diabetes mellitus: subanalysis of the TELEREH-HF randomized clinical trial
Source: Cardiovasc Diabetol. 2021 May 13;20:106. doi: 10.1186/s12933-021-01292-9 (PMC8120915; doi:10.1186/s12933-021-01292-9)
Supplement: Supplementary file 1 — Additional file 1: Table S1. TELEREH-HF Exercise Training Model [21]. Table S2. Inclusion and Exclusion Criteria for TELEREH-HF trial [20, 21]. Table S3. Baseline characteristics of excluded vs studied patients. Table S4. Baseline characteristics of studied patients with and without diabetes. Table S5. Baseline parameters of cardiopulmonary capacity. [file 12933_2021_1292_MOESM1_ESM.docx]

**Table S1. TELEREH-HF Exercise Training Model [21]**

| Type of exercise training | Exercise prescription |
| --- | --- |
| Aerobic endurance training | Devices: Nordic walking poles Training session consists of: 1.Warm-up: breathing and light resistance exercises using poles for Nordic walking; duration 5–10 min 2.Interval Nordic walking training Intensity: 40-70% of heart rate reserve, perceived exertion level—score of 11-12 on the Borg scale Duration: start at 10 min/session/day a 15 min/session/day b 20 min/session/day c gradually increased to 30–45 min/session/day 3.Cool down: relaxation, breathing exercise; duration 5 min Frequency: 1 session/day |
| Respiratory muscle training | Devices: Train Air software - during the initial stage at the hospital Threshold Inspiratory Muscle Trainer - during the basic stage at home Intensity: start at 30% of the maximal inspiratory mouth pressure (PImax) and readjusted to a maximum of 60% (if possible) Duration: minimum 5-10 minutes/day maximum 20-30 minutes/day; Frequency: 3-5 times/ throughout the day |
| Resistance and strength training | Devices: Thera Band - yellow color Intensity: 5-10 repetitions of each of the seven exercises Duration: gradually increased 5-10-15 minutes/day Frequency: 1 session/ day |

Duration of aerobic endurance training depended on the functional capacity in baseline cardiopulmonary exercise test:

1. baseline VO_2_ peak below 10 mL/kg/min.
2. baseline VO_2_ peak 10–18 mL/kg/min.
3. baseline VO_2_ peak over 18 mL/kg/min.

**Table S2. Inclusion and Exclusion Criteria for TELEREH-HF trial [20, 21].**

| **Inclusion criteria** |
| --- |
| Clinically stable patients  New York Heart Association [NYHA] class I, II or III  Left ventricular ejection fraction [LVEF] ≤ 40%  Within 6 months after hospitalization |
| **Exclusion Criteria** |
| None of the following condition may exist at randomization:  - NYHA class IV  - unstable angina  - unstable clinical status  - a history of acute coronary syndrome within the last forty days in patients with LVEF ≤ 35%  - percutaneous angioplasty within the last 2 weeks  - coronary artery bypass grafting within the last 3 months  - initiation of CRT-P or CRT-D or ICD or PM within the last six weeks  - lack of ICD, CRT-P or CRT-D or PM therapy despite the indications for implantation  according to ESC guidelines  - intracardiac thrombus  - rest heart rate >90/min  - tachypnoe >20 breaths per minute  - symptomatic and/or exercise-induced cardiac arrhythmia or conduction disturbances  - acute myocarditis and/or pericarditis  - valvular or congenital heart disease requiring surgical treatment  - hypertrophic cardiomyopathy  - severe pulmonary disease  - uncontrolled hypertension  - anemia (hemoglobin <11.0 g/dL)  - physical disability related to severe musculoskeletal or neurological problems  - recent embolism  - thrombophlebitis  - acute or chronic inflammatory disease  - acute or chronic decompensated non-cardiac diseases (thyreotoxicosis, uncontrolled diabetes)  - active malignant neoplastic diseases with survival prognosis below 2 – 5 years  - orthotropic heart transplant in anamnesis  - presence of an implanted left ventricualr assist device or biventricular assist device  - aortic aneurysm  - severe psychiatric disorder  - patient’s refusal to participate |

ESC - European Society of Cardiology, LVEF - left ventricular ejection fraction, NYHA - New York Heart Association, CRT-P - cardiac resynchronization therapy, CRT-D - cardiac resynchronization therapy and implantable cardioverter-defibrillator, ICD - implantable cardioverter-defibrillator, PM – pacemaker

**Table S3. Baseline characteristics of excluded vs studied patients.**

|  | Excluded patients | Analyzed patients | p |
| --- | --- | --- | --- |
|  | 68 patients (8%) | 782 patients (92%) |  |
| Males. n (%) | 55 (80.9) | 698 (89.3) | 0.037 |
| Age (years). mean ± SD | 66.7 ± 9.4 | 62.0 ± 10.5 | <0.001 |
| BMI | 28.3 ± 4,6 | 29.0 ± 4.9 | 0.247 |
| Left Ventricular Ejection Fraction (%). mean ± SD | 30.0 ± 7.9 | 30.7 ± 6.9 | 0.426 |
| Atrial fibrillation or atrial flutter. n (%) | 14 (20.6) | 145 (18.5) | 0.678 |
| Etiology of heart failure. n (%) |  |  |  |
| Ischaemic | 49 (72.1) | 506 (64.7) | 0.222 |
| Non-ischeamic | 19 (27.9) | 276 (35.3) |  |
| **Previous medical history n (%)** |  |  |  |
| Coronary artery disease | 49 (72.1) | 502 (64.2) | 0.193 |
| Myocardial infarction | 46 (67.6) | 447 (57.2) | 0.093 |
| Angioplasty | 32 (47.1) | 364 (46.5) | 0.935 |
| Coronary artery bypass grafting | 18 (26.5) | 122 (15.6) | 0.021 |
| Hypertension | 52 (76.5) | 482 (61.6) | 0.015 |
| Stroke | 10 (14.7) | 51 (6.5) | 0.023 |
| Chronic kidney disease | 18 (26.5) | 131 (16.7) | 0.043 |
| Hyperlipidemia | 32 (47.1) | 364 (46.6) | 0.935 |
| **Functional status** |  |  |  |
| NYHA I. n (%) | 4 (5.9) | 100 (12.8) | <0.001 |
| NYHA II. n (%) | 36 (52.9) | 541 (69.2) |  |
| NYHA III. n (%) | 28 (41.2) | 141 (18.0) |  |
| **Treatment** |  |  |  |
| Beta-blocker | 67 (98.5) | 758 (96.9) | 0.714 |
| ACEI/ARB | 62 (91.2) | 731 (93.5) | 0.447 |
| Digoxin | 7 (10.3) | 97 (12.4) | 0.611 |
| Loop diuretics | 59 (86.8) | 591 (75.6) | 0.037 |
| Spironolactone/eplerenone | 56 (82.3) | 643 (82.2) | 0.979 |
| Aspirin/clopidogrel | 42 (61.8) | 443 (56.6) | 0.414 |
| Anticoagulants | 19 (27.9) | 234 (29.9) | 0.732 |
| Statins | 57 (83.8) | 639 (81.7) | 0.665 |
| CIEDs | 56 (82.3) | 626 (80.1) | 0.647 |
| Implantable cardioverter-defibrillator | 35 (62.5) | 396 (63.3) | 0.684 |
| CRT-P | 0 | 8 (1.3) |  |
| CRT-D | 21 (37.5) | 215 (34.4) |  |

Reasons for lack of both CPETs:

- in HCTR group (40 patients): lack of second hospitalization and CPET with continuation of Follow-up (27 patients), resignation (9 patients), decompensation of heart failure (1 patient), infective endocarditis (1 patient), death (2 patients).
- In UC group: resignation (16 patients), decompensation of heart failure (8 patients), ventricular arrhythmia (1 patient), death (2 patients).

**Table S4. Baseline characteristics of studied patients with and without diabetes.**

|  |  |  |  |
| --- | --- | --- | --- |
|  | DM, n=266 | nonDM, n=516 | P- |
| Males. n (%) | 245 (92.1) | 453 (87.8) | 0.065 |
| Age (years). mean ± SD | 64.2 ± 8.1 | 60.8 ± 11.4 | <0.001 |
| BMI (kg/m2). mean ± SD | 30.6 ± 4.9 | 28.1 ± 4.7 | <0.001 |
| Left Ventricular Ejection Fraction (%). mean ± SD | 30.3 ± 6.6 | 30.9 ± 7.1 | 0.293 |
| Atrial fibrillation or atrial flutter. n (%) | 59 (22.2) | 86 (16.7) | 0.060 |
| Etiology of heart failure. n (%) |  |  |  |
| Ischaemic | 190 (71.4) | 316 (61.2) | 0.005 |
| Non-ischeamic | 76 (28.6) | 200 (38.8) |  |
| **Previous medical history n (%)** |  |  |  |
| Coronary artery disease | 187 (70.3) | 315 (61.0) | 0.010 |
| Myocardial infarction | 168 (63.2) | 279 (54.1) | 0.015 |
| Angioplasty | 134 (50.4) | 230 (44.6) | 0.123 |
| Coronary artery bypass grafting | 57 (21.4) | 65 (12.6) | 0.001 |
| Hypertension | 196 (73.7) | 286 (55.4) | <0.001 |
| Stroke | 24 (9.0) | 27 (5.2) | 0.042 |
| Chronic kidney disease | 72 (27.1) | 59 (11.4) | <0.001 |
| Hyperlipidemia | 125 (47.0) | 239 (46.3) | 0.858 |
| **Functional status** |  |  |  |
| NYHA I. n (%) | 18 (6.8) | 82 (15.9) | <0.001 |
| NYHA II. n (%) | 175 (65.8) | 366 (70.9) |  |
| NYHA III. n (%) | 73 (27.4) | 68 (13.2) |  |
| **Treatment** |  |  |  |
| Beta-blocker | 264 (99.2) | 494 (95.7) | 0.007 |
| ACEI/ARB | 246 (92.5) | 485 (94.0) | 0.417 |
| Digoxin | 47 (17.7) | 50 (9.7) | 0.001 |
| Loop diuretics | 225 (84.6) | 366 (70.9) | <0.001 |
| Spironolactone/eplerenone | 211 (79.3) | 432 (83.7) | 0.127 |
| Aspirin/clopidogrel | 151 (56.8) | 292 (56.6) | 0.962 |
| Anticoagulants | 93 (35.0) | 141 (27.3) | 0.027 |
| Statins | 232 (87.2) | 407 (78.9) | 0.004 |
| CIEDs | 223 (83.8) | 403 (78.1) | 0.057 |
| Implantable cardioverter-defibrillator | 131 (58.7) | 265 (65.8) | 0.100 |
| CRT-P | 1 (0.4) | 7 (1.7) |  |
| CRT-D | 89 (39.9) | 126 (31.3) |  |

**Table S5. Baseline parameters of cardiopulmonary capacity**

| **CPET parameters** | DM, n=266 | nonDM, n=516 | p |
| --- | --- | --- | --- |
| **Exercise time (s)** | 332 ± 163 | 414 ± 187 | <0.001 |
| **VO_2_ peak (mL/min/kg)** | 15.2 ± 4.8 | 17.9 ± 6.1 | <0.001 |
| **VCO_2_ peak (L**/min) | 1.33 ± 0.57 | 1.53 ± 0.68 | <0.001 |
| **Percent-predicted VO_2_ (%)** | 48.1 ± 17.2 | 59.2 ± 21.3 | <0.001 |
| **VAT (ml/kg/min):** | 13.8 ± 5.0 | 15.7 ± 5.7 | <0.001 |
| **Ventilation at rest (l/min)** | 13.1 ± 4.3 | 12.9 ± 4.8 | 0.451 |
| **Ventilation on peak exercise (l/min)** | 46.2 ± 16.0 | 51.1 ± 19.0 | <0.001 |
| **Breathing rate at rest(1/min)** | 18.9 ± 4.8 | 18.9 ± 4.8 | 0.977 |
| **Breathing rate on peak exercise (1/min)** | 28.9 ± 5.8 | 29.7 ± 6.7 | 0.071 |
| **VE/VO_2_ slope** | 30.0 ± 12.0 | 31.1 ± 11.2 | 0.220 |
| **VE/VCO_2_ slope** | 31.7 ± 10.5 | 29.7 ± 10.6 | 0.013 |
